# Supplementary material for: Modeling glioblastoma heterogeneity as a dynamic network of cell states
Source: Mol Syst Biol. 2021 Sep 16;17(9):e10105. doi: 10.15252/msb.202010105 (PMC8444284; doi:10.15252/msb.202010105)
Supplement: Supplementary file 5 — Source Data for Figure 3 [file MSB-17-e10105-s001.zip › Figure3A_sourcedata/GSEA_3065/nosigdb_state2.GseaPreranked.1622718419215/DOROSZKO_U3180MG_CLUSTER_3.html]

Details for gene set DOROSZKO\_U3180MG\_CLUSTER\_3[GSEA]

|  || Dataset | state2 |
| Phenotype | NoPhenotypeAvailable |
| Upregulated in class | na\_pos |
| GeneSet | DOROSZKO\_U3180MG\_CLUSTER\_3 |
| Enrichment Score (ES) | 0.8626696 |
| Normalized Enrichment Score (NES) | 3.3046262 |
| Nominal p-value | 0.0 |
| FDR q-value | 0.0 |
| FWER p-Value | 0.0 |
Table: GSEA Results Summary

  

Fig 1: Enrichment plot: DOROSZKO\_U3180MG\_CLUSTER\_3      
 Profile of the Running ES Score & Positions of GeneSet Members on the Rank Ordered List

  

| PROBE | GENE SYMBOL | GENE\_TITLE | RANK IN GENE LIST | RANK METRIC SCORE | RUNNING ES | CORE ENRICHMENT || 1 | HIST1H4C |  |  | 0 | 1.309 | 0.0112 | Yes |
| 2 | PCLAF |  |  | 2 | 0.852 | 0.0184 | Yes |
| 3 | HIST1H1D |  |  | 3 | 0.842 | 0.0257 | Yes |
| 4 | HMGB2 |  |  | 4 | 0.823 | 0.0327 | Yes |
| 5 | TYMS |  |  | 5 | 0.792 | 0.0395 | Yes |
| 6 | CENPU |  |  | 6 | 0.763 | 0.0461 | Yes |
| 7 | H2AFZ |  |  | 8 | 0.735 | 0.0523 | Yes |
| 8 | UBE2C |  |  | 9 | 0.704 | 0.0583 | Yes |
| 9 | H2AFX |  |  | 10 | 0.675 | 0.0641 | Yes |
| 10 | DHFR |  |  | 11 | 0.644 | 0.0696 | Yes |
| 11 | UBE2T |  |  | 12 | 0.643 | 0.0752 | Yes |
| 12 | MCM7 |  |  | 13 | 0.626 | 0.0805 | Yes |
| 13 | MYBL2 |  |  | 14 | 0.600 | 0.0857 | Yes |
| 14 | TOP2A |  |  | 15 | 0.596 | 0.0908 | Yes |
| 15 | PCNA |  |  | 16 | 0.595 | 0.0959 | Yes |
| 16 | MKI67 |  |  | 17 | 0.590 | 0.1010 | Yes |
| 17 | CDK1 |  |  | 18 | 0.577 | 0.1059 | Yes |
| 18 | MAD2L1 |  |  | 20 | 0.563 | 0.1106 | Yes |
| 19 | ATAD2 |  |  | 21 | 0.563 | 0.1155 | Yes |
| 20 | RRM2 |  |  | 22 | 0.556 | 0.1202 | Yes |
| 21 | CKS1B |  |  | 23 | 0.554 | 0.1250 | Yes |
| 22 | DUT |  |  | 24 | 0.552 | 0.1297 | Yes |
| 23 | NUDT1 |  |  | 26 | 0.541 | 0.1343 | Yes |
| 24 | CLSPN |  |  | 27 | 0.536 | 0.1389 | Yes |
| 25 | CKS2 |  |  | 28 | 0.532 | 0.1434 | Yes |
| 26 | TPX2 |  |  | 29 | 0.531 | 0.1480 | Yes |
| 27 | ESCO2 |  |  | 30 | 0.530 | 0.1525 | Yes |
| 28 | DEK |  |  | 31 | 0.526 | 0.1571 | Yes |
| 29 | FANCI |  |  | 32 | 0.521 | 0.1615 | Yes |
| 30 | CENPF |  |  | 34 | 0.518 | 0.1658 | Yes |
| 31 | RPA3 |  |  | 36 | 0.511 | 0.1701 | Yes |
| 32 | GINS2 |  |  | 37 | 0.506 | 0.1745 | Yes |
| 33 | SMC2 |  |  | 39 | 0.493 | 0.1786 | Yes |
| 34 | ORC6 |  |  | 40 | 0.493 | 0.1828 | Yes |
| 35 | DBF4 |  |  | 41 | 0.492 | 0.1870 | Yes |
| 36 | SMC4 |  |  | 42 | 0.489 | 0.1912 | Yes |
| 37 | TMPO |  |  | 43 | 0.489 | 0.1954 | Yes |
| 38 | CENPK |  |  | 44 | 0.488 | 0.1996 | Yes |
| 39 | ZWINT |  |  | 45 | 0.488 | 0.2038 | Yes |
| 40 | PBK |  |  | 46 | 0.484 | 0.2080 | Yes |
| 41 | NUSAP1 |  |  | 47 | 0.483 | 0.2121 | Yes |
| 42 | ANP32B |  |  | 48 | 0.474 | 0.2162 | Yes |
| 43 | KPNA2 |  |  | 50 | 0.472 | 0.2201 | Yes |
| 44 | MCM3 |  |  | 51 | 0.464 | 0.2241 | Yes |
| 45 | RFC2 |  |  | 53 | 0.460 | 0.2279 | Yes |
| 46 | CENPM |  |  | 54 | 0.460 | 0.2319 | Yes |
| 47 | CHAF1A |  |  | 55 | 0.457 | 0.2358 | Yes |
| 48 | TK1 |  |  | 56 | 0.456 | 0.2397 | Yes |
| 49 | UHRF1 |  |  | 57 | 0.454 | 0.2436 | Yes |
| 50 | SGO1 |  |  | 58 | 0.449 | 0.2475 | Yes |
| 51 | E2F1 |  |  | 59 | 0.446 | 0.2513 | Yes |
| 52 | NCAPG |  |  | 60 | 0.445 | 0.2551 | Yes |
| 53 | BIRC5 |  |  | 61 | 0.443 | 0.2589 | Yes |
| 54 | SIVA1 |  |  | 62 | 0.439 | 0.2627 | Yes |
| 55 | GMNN |  |  | 64 | 0.435 | 0.2663 | Yes |
| 56 | RANBP1 |  |  | 65 | 0.430 | 0.2700 | Yes |
| 57 | HIST1H1E |  |  | 66 | 0.429 | 0.2737 | Yes |
| 58 | FBXO5 |  |  | 67 | 0.420 | 0.2773 | Yes |
| 59 | LMNB1 |  |  | 69 | 0.417 | 0.2808 | Yes |
| 60 | EZH2 |  |  | 70 | 0.417 | 0.2843 | Yes |
| 61 | CENPH |  |  | 71 | 0.412 | 0.2879 | Yes |
| 62 | RRM1 |  |  | 72 | 0.412 | 0.2914 | Yes |
| 63 | LSM4 |  |  | 73 | 0.410 | 0.2949 | Yes |
| 64 | SNRPD1 |  |  | 74 | 0.408 | 0.2984 | Yes |
| 65 | LSM3 |  |  | 75 | 0.407 | 0.3019 | Yes |
| 66 | ASF1B |  |  | 76 | 0.407 | 0.3054 | Yes |
| 67 | SGO2 |  |  | 77 | 0.406 | 0.3089 | Yes |
| 68 | MCM4 |  |  | 79 | 0.399 | 0.3122 | Yes |
| 69 | PIMREG |  |  | 80 | 0.398 | 0.3156 | Yes |
| 70 | TUBB |  |  | 81 | 0.397 | 0.3190 | Yes |
| 71 | H2AFV |  |  | 82 | 0.397 | 0.3224 | Yes |
| 72 | MCM5 |  |  | 83 | 0.394 | 0.3258 | Yes |
| 73 | KIF4A |  |  | 84 | 0.393 | 0.3292 | Yes |
| 74 | KIF15 |  |  | 86 | 0.392 | 0.3324 | Yes |
| 75 | CENPN |  |  | 87 | 0.390 | 0.3358 | Yes |
| 76 | HNRNPD |  |  | 88 | 0.390 | 0.3391 | Yes |
| 77 | GTSE1 |  |  | 89 | 0.389 | 0.3425 | Yes |
| 78 | RAD51AP1 |  |  | 90 | 0.388 | 0.3458 | Yes |
| 79 | ALYREF |  |  | 91 | 0.382 | 0.3491 | Yes |
| 80 | BRCA1 |  |  | 92 | 0.381 | 0.3523 | Yes |
| 81 | PHF19 |  |  | 93 | 0.380 | 0.3556 | Yes |
| 82 | DTYMK |  |  | 94 | 0.379 | 0.3589 | Yes |
| 83 | CDC45 |  |  | 95 | 0.377 | 0.3621 | Yes |
| 84 | MND1 |  |  | 96 | 0.376 | 0.3653 | Yes |
| 85 | CDKN3 |  |  | 97 | 0.376 | 0.3685 | Yes |
| 86 | MCM10 |  |  | 100 | 0.373 | 0.3715 | Yes |
| 87 | CDCA5 |  |  | 101 | 0.373 | 0.3747 | Yes |
| 88 | GGCT |  |  | 102 | 0.373 | 0.3779 | Yes |
| 89 | DNAJC9 |  |  | 103 | 0.373 | 0.3811 | Yes |
| 90 | SPC25 |  |  | 104 | 0.372 | 0.3843 | Yes |
| 91 | CDC6 |  |  | 106 | 0.372 | 0.3874 | Yes |
| 92 | FEN1 |  |  | 107 | 0.371 | 0.3906 | Yes |
| 93 | CDT1 |  |  | 108 | 0.368 | 0.3937 | Yes |
| 94 | CACYBP |  |  | 109 | 0.366 | 0.3969 | Yes |
| 95 | SLBP |  |  | 112 | 0.357 | 0.3997 | Yes |
| 96 | HNRNPAB |  |  | 113 | 0.357 | 0.4028 | Yes |
| 97 | CENPX |  |  | 114 | 0.356 | 0.4058 | Yes |
| 98 | NDC80 |  |  | 115 | 0.354 | 0.4089 | Yes |
| 99 | CENPV |  |  | 116 | 0.353 | 0.4119 | Yes |
| 100 | KIF23 |  |  | 117 | 0.351 | 0.4149 | Yes |
| 101 | VRK1 |  |  | 118 | 0.350 | 0.4179 | Yes |
| 102 | POP7 |  |  | 119 | 0.347 | 0.4209 | Yes |
| 103 | POLD3 |  |  | 120 | 0.347 | 0.4239 | Yes |
| 104 | DNMT1 |  |  | 121 | 0.345 | 0.4268 | Yes |
| 105 | ASPM |  |  | 122 | 0.342 | 0.4298 | Yes |
| 106 | TUBB4B |  |  | 125 | 0.340 | 0.4325 | Yes |
| 107 | RFC4 |  |  | 126 | 0.339 | 0.4354 | Yes |
| 108 | CCDC34 |  |  | 127 | 0.339 | 0.4383 | Yes |
| 109 | TACC3 |  |  | 130 | 0.334 | 0.4409 | Yes |
| 110 | CDK4 |  |  | 131 | 0.333 | 0.4438 | Yes |
| 111 | SNRPB |  |  | 132 | 0.332 | 0.4466 | Yes |
| 112 | MELK |  |  | 133 | 0.332 | 0.4495 | Yes |
| 113 | POC1A |  |  | 134 | 0.332 | 0.4523 | Yes |
| 114 | CCNA2 |  |  | 135 | 0.331 | 0.4552 | Yes |
| 115 | ANP32E |  |  | 136 | 0.330 | 0.4580 | Yes |
| 116 | PA2G4 |  |  | 137 | 0.329 | 0.4608 | Yes |
| 117 | USP1 |  |  | 138 | 0.329 | 0.4637 | Yes |
| 118 | RBBP8 |  |  | 141 | 0.325 | 0.4662 | Yes |
| 119 | MIS18A |  |  | 142 | 0.322 | 0.4690 | Yes |
| 120 | ARHGAP11A |  |  | 145 | 0.321 | 0.4715 | Yes |
| 121 | RAD21 |  |  | 147 | 0.316 | 0.4741 | Yes |
| 122 | DTL |  |  | 148 | 0.311 | 0.4768 | Yes |
| 123 | MCM6 |  |  | 149 | 0.311 | 0.4795 | Yes |
| 124 | CDCA2 |  |  | 150 | 0.309 | 0.4821 | Yes |
| 125 | CKAP2 |  |  | 151 | 0.308 | 0.4848 | Yes |
| 126 | TPRKB |  |  | 153 | 0.307 | 0.4873 | Yes |
| 127 | KIF22 |  |  | 154 | 0.306 | 0.4899 | Yes |
| 128 | PSMC3IP |  |  | 159 | 0.305 | 0.4921 | Yes |
| 129 | CDCA3 |  |  | 160 | 0.304 | 0.4947 | Yes |
| 130 | HELLS |  |  | 161 | 0.304 | 0.4973 | Yes |
| 131 | TMEM106C |  |  | 162 | 0.303 | 0.4999 | Yes |
| 132 | AURKB |  |  | 163 | 0.303 | 0.5025 | Yes |
| 133 | LMNB2 |  |  | 169 | 0.298 | 0.5045 | Yes |
| 134 | CMC2 |  |  | 170 | 0.297 | 0.5070 | Yes |
| 135 | DCTPP1 |  |  | 172 | 0.296 | 0.5095 | Yes |
| 136 | BTG3 |  |  | 173 | 0.295 | 0.5120 | Yes |
| 137 | PSIP1 |  |  | 175 | 0.294 | 0.5144 | Yes |
| 138 | ITGB3BP |  |  | 177 | 0.294 | 0.5168 | Yes |
| 139 | CARHSP1 |  |  | 178 | 0.294 | 0.5194 | Yes |
| 140 | ACAT2 |  |  | 180 | 0.294 | 0.5218 | Yes |
| 141 | DDX39A |  |  | 183 | 0.293 | 0.5241 | Yes |
| 142 | CDCA4 |  |  | 184 | 0.292 | 0.5266 | Yes |
| 143 | BARD1 |  |  | 185 | 0.292 | 0.5291 | Yes |
| 144 | PDCD5 |  |  | 186 | 0.291 | 0.5316 | Yes |
| 145 | NUF2 |  |  | 188 | 0.287 | 0.5339 | Yes |
| 146 | FAM111B |  |  | 191 | 0.285 | 0.5361 | Yes |
| 147 | HAUS1 |  |  | 192 | 0.285 | 0.5386 | Yes |
| 148 | PARP1 |  |  | 194 | 0.284 | 0.5409 | Yes |
| 149 | DEPDC1 |  |  | 196 | 0.283 | 0.5432 | Yes |
| 150 | SMC3 |  |  | 197 | 0.283 | 0.5457 | Yes |
| 151 | BANF1 |  |  | 198 | 0.283 | 0.5481 | Yes |
| 152 | MAGOHB |  |  | 199 | 0.282 | 0.5505 | Yes |
| 153 | HMGB3 |  |  | 200 | 0.282 | 0.5529 | Yes |
| 154 | NCAPG2 |  |  | 201 | 0.282 | 0.5553 | Yes |
| 155 | FOXM1 |  |  | 202 | 0.282 | 0.5578 | Yes |
| 156 | PAFAH1B3 |  |  | 203 | 0.282 | 0.5602 | Yes |
| 157 | C3orf14 |  |  | 204 | 0.279 | 0.5626 | Yes |
| 158 | HNRNPF |  |  | 205 | 0.279 | 0.5650 | Yes |
| 159 | TEX30 |  |  | 206 | 0.279 | 0.5674 | Yes |
| 160 | HJURP |  |  | 209 | 0.277 | 0.5695 | Yes |
| 161 | HSPB11 |  |  | 210 | 0.277 | 0.5719 | Yes |
| 162 | H1FX |  |  | 212 | 0.276 | 0.5742 | Yes |
| 163 | NME1 |  |  | 214 | 0.274 | 0.5764 | Yes |
| 164 | C19orf48 |  |  | 215 | 0.274 | 0.5788 | Yes |
| 165 | DSN1 |  |  | 216 | 0.273 | 0.5811 | Yes |
| 166 | IDH2 |  |  | 218 | 0.272 | 0.5833 | Yes |
| 167 | ATAD5 |  |  | 222 | 0.269 | 0.5853 | Yes |
| 168 | UBA2 |  |  | 223 | 0.268 | 0.5876 | Yes |
| 169 | CHEK1 |  |  | 224 | 0.266 | 0.5899 | Yes |
| 170 | CBX5 |  |  | 225 | 0.265 | 0.5922 | Yes |
| 171 | SNRPD3 |  |  | 226 | 0.265 | 0.5944 | Yes |
| 172 | MCM2 |  |  | 228 | 0.262 | 0.5966 | Yes |
| 173 | GNG4 |  |  | 229 | 0.262 | 0.5988 | Yes |
| 174 | SHCBP1 |  |  | 231 | 0.262 | 0.6010 | Yes |
| 175 | EIF4EBP1 |  |  | 232 | 0.261 | 0.6032 | Yes |
| 176 | WDR34 |  |  | 235 | 0.260 | 0.6052 | Yes |
| 177 | SNRPF |  |  | 237 | 0.259 | 0.6073 | Yes |
| 178 | SUZ12 |  |  | 239 | 0.258 | 0.6094 | Yes |
| 179 | CCDC167 |  |  | 243 | 0.255 | 0.6113 | Yes |
| 180 | SMC1A |  |  | 249 | 0.253 | 0.6129 | Yes |
| 181 | RALY |  |  | 250 | 0.253 | 0.6150 | Yes |
| 182 | KIF2C |  |  | 252 | 0.251 | 0.6171 | Yes |
| 183 | CKLF |  |  | 254 | 0.249 | 0.6191 | Yes |
| 184 | SPC24 |  |  | 256 | 0.248 | 0.6211 | Yes |
| 185 | MIS18BP1 |  |  | 257 | 0.246 | 0.6232 | Yes |
| 186 | MRPL18 |  |  | 258 | 0.246 | 0.6254 | Yes |
| 187 | LIG1 |  |  | 259 | 0.246 | 0.6275 | Yes |
| 188 | RAD51C |  |  | 260 | 0.246 | 0.6296 | Yes |
| 189 | PEG10 |  |  | 261 | 0.246 | 0.6317 | Yes |
| 190 | TUBG1 |  |  | 262 | 0.246 | 0.6338 | Yes |
| 191 | SAC3D1 |  |  | 263 | 0.246 | 0.6359 | Yes |
| 192 | MSH2 |  |  | 266 | 0.244 | 0.6378 | Yes |
| 193 | PHGDH |  |  | 271 | 0.243 | 0.6394 | Yes |
| 194 | TIMELESS |  |  | 272 | 0.243 | 0.6415 | Yes |
| 195 | PSMC3 |  |  | 275 | 0.242 | 0.6434 | Yes |
| 196 | CHCHD3 |  |  | 277 | 0.242 | 0.6453 | Yes |
| 197 | SAE1 |  |  | 278 | 0.242 | 0.6474 | Yes |
| 198 | CCT5 |  |  | 279 | 0.242 | 0.6495 | Yes |
| 199 | HAUS8 |  |  | 280 | 0.242 | 0.6516 | Yes |
| 200 | CMSS1 |  |  | 281 | 0.241 | 0.6536 | Yes |
| 201 | TRIP13 |  |  | 286 | 0.237 | 0.6552 | Yes |
| 202 | ACTL6A |  |  | 287 | 0.237 | 0.6572 | Yes |
| 203 | CCNE2 |  |  | 288 | 0.236 | 0.6593 | Yes |
| 204 | YEATS4 |  |  | 293 | 0.235 | 0.6608 | Yes |
| 205 | NCAPD3 |  |  | 294 | 0.234 | 0.6628 | Yes |
| 206 | NME4 |  |  | 295 | 0.234 | 0.6649 | Yes |
| 207 | PIN1 |  |  | 296 | 0.234 | 0.6669 | Yes |
| 208 | RFC5 |  |  | 297 | 0.233 | 0.6689 | Yes |
| 209 | BAZ1B |  |  | 303 | 0.230 | 0.6703 | Yes |
| 210 | CIP2A |  |  | 304 | 0.230 | 0.6723 | Yes |
| 211 | COX5A |  |  | 308 | 0.229 | 0.6739 | Yes |
| 212 | BUB3 |  |  | 311 | 0.228 | 0.6756 | Yes |
| 213 | POLR2I |  |  | 315 | 0.227 | 0.6772 | Yes |
| 214 | RAD18 |  |  | 317 | 0.227 | 0.6791 | Yes |
| 215 | TCF19 |  |  | 318 | 0.226 | 0.6810 | Yes |
| 216 | SSRP1 |  |  | 319 | 0.226 | 0.6830 | Yes |
| 217 | HNRNPA3 |  |  | 320 | 0.226 | 0.6849 | Yes |
| 218 | DIAPH3 |  |  | 321 | 0.226 | 0.6868 | Yes |
| 219 | CKAP2L |  |  | 327 | 0.224 | 0.6882 | Yes |
| 220 | MXD3 |  |  | 328 | 0.224 | 0.6901 | Yes |
| 221 | TMEM97 |  |  | 331 | 0.223 | 0.6918 | Yes |
| 222 | POLE3 |  |  | 332 | 0.223 | 0.6937 | Yes |
| 223 | RACGAP1 |  |  | 333 | 0.222 | 0.6956 | Yes |
| 224 | NCAPH |  |  | 336 | 0.222 | 0.6973 | Yes |
| 225 | NDUFA6 |  |  | 338 | 0.222 | 0.6991 | Yes |
| 226 | PKMYT1 |  |  | 342 | 0.220 | 0.7006 | Yes |
| 227 | MRPL17 |  |  | 344 | 0.218 | 0.7024 | Yes |
| 228 | COMMD4 |  |  | 345 | 0.218 | 0.7043 | Yes |
| 229 | NUDC |  |  | 346 | 0.218 | 0.7062 | Yes |
| 230 | HIRIP3 |  |  | 347 | 0.217 | 0.7080 | Yes |
| 231 | KPNB1 |  |  | 350 | 0.216 | 0.7097 | Yes |
| 232 | COA1 |  |  | 353 | 0.216 | 0.7113 | Yes |
| 233 | LSM8 |  |  | 354 | 0.215 | 0.7131 | Yes |
| 234 | MASTL |  |  | 357 | 0.215 | 0.7148 | Yes |
| 235 | GPN3 |  |  | 360 | 0.214 | 0.7164 | Yes |
| 236 | PPIH |  |  | 362 | 0.214 | 0.7181 | Yes |
| 237 | GLO1 |  |  | 363 | 0.214 | 0.7199 | Yes |
| 238 | POLA2 |  |  | 366 | 0.212 | 0.7215 | Yes |
| 239 | RPA2 |  |  | 367 | 0.212 | 0.7233 | Yes |
| 240 | MGME1 |  |  | 370 | 0.211 | 0.7249 | Yes |
| 241 | CENPW |  |  | 373 | 0.211 | 0.7265 | Yes |
| 242 | ZWILCH |  |  | 374 | 0.210 | 0.7283 | Yes |
| 243 | H2AFY |  |  | 376 | 0.209 | 0.7300 | Yes |
| 244 | KNL1 |  |  | 379 | 0.209 | 0.7316 | Yes |
| 245 | MSH6 |  |  | 380 | 0.209 | 0.7334 | Yes |
| 246 | HAT1 |  |  | 381 | 0.209 | 0.7351 | Yes |
| 247 | CDK2 |  |  | 382 | 0.208 | 0.7369 | Yes |
| 248 | AP2S1 |  |  | 385 | 0.208 | 0.7385 | Yes |
| 249 | CSE1L |  |  | 391 | 0.204 | 0.7397 | Yes |
| 250 | UQCC2 |  |  | 393 | 0.202 | 0.7413 | Yes |
| 251 | SLF1 |  |  | 394 | 0.202 | 0.7431 | Yes |
| 252 | DSCC1 |  |  | 395 | 0.202 | 0.7448 | Yes |
| 253 | UNG |  |  | 398 | 0.200 | 0.7463 | Yes |
| 254 | CCT4 |  |  | 405 | 0.199 | 0.7473 | Yes |
| 255 | BRIP1 |  |  | 426 | 0.194 | 0.7467 | Yes |
| 256 | BRCA2 |  |  | 427 | 0.193 | 0.7484 | Yes |
| 257 | PSMG1 |  |  | 428 | 0.193 | 0.7501 | Yes |
| 258 | BCL7C |  |  | 429 | 0.193 | 0.7517 | Yes |
| 259 | PXMP2 |  |  | 430 | 0.193 | 0.7534 | Yes |
| 260 | COPS3 |  |  | 437 | 0.192 | 0.7544 | Yes |
| 261 | TEDC1 |  |  | 438 | 0.192 | 0.7560 | Yes |
| 262 | FANCD2 |  |  | 447 | 0.191 | 0.7568 | Yes |
| 263 | FAM111A |  |  | 448 | 0.191 | 0.7584 | Yes |
| 264 | MZT2B |  |  | 450 | 0.190 | 0.7599 | Yes |
| 265 | XRCC2 |  |  | 455 | 0.190 | 0.7611 | Yes |
| 266 | EXOSC8 |  |  | 456 | 0.189 | 0.7627 | Yes |
| 267 | UBR7 |  |  | 458 | 0.189 | 0.7642 | Yes |
| 268 | MAD2L2 |  |  | 460 | 0.189 | 0.7657 | Yes |
| 269 | XRCC5 |  |  | 461 | 0.189 | 0.7674 | Yes |
| 270 | RTKN2 |  |  | 463 | 0.188 | 0.7689 | Yes |
| 271 | DCK |  |  | 465 | 0.187 | 0.7704 | Yes |
| 272 | GINS1 |  |  | 470 | 0.185 | 0.7715 | Yes |
| 273 | DERA |  |  | 471 | 0.185 | 0.7731 | Yes |
| 274 | CENPQ |  |  | 472 | 0.185 | 0.7747 | Yes |
| 275 | MEA1 |  |  | 476 | 0.184 | 0.7759 | Yes |
| 276 | HDAC2 |  |  | 479 | 0.184 | 0.7773 | Yes |
| 277 | MRPS26 |  |  | 485 | 0.183 | 0.7783 | Yes |
| 278 | PGAM1 |  |  | 487 | 0.183 | 0.7797 | Yes |
| 279 | MRM2 |  |  | 495 | 0.182 | 0.7805 | Yes |
| 280 | SKA1 |  |  | 497 | 0.182 | 0.7820 | Yes |
| 281 | YWHAQ |  |  | 498 | 0.182 | 0.7835 | Yes |
| 282 | DEPDC1B |  |  | 505 | 0.179 | 0.7844 | Yes |
| 283 | RNASEH2B |  |  | 507 | 0.179 | 0.7858 | Yes |
| 284 | PRC1 |  |  | 514 | 0.178 | 0.7867 | Yes |
| 285 | LRR1 |  |  | 516 | 0.178 | 0.7881 | Yes |
| 286 | SNRNP25 |  |  | 525 | 0.177 | 0.7887 | Yes |
| 287 | SUPT16H |  |  | 529 | 0.176 | 0.7899 | Yes |
| 288 | CEP57 |  |  | 531 | 0.176 | 0.7913 | Yes |
| 289 | CTNNAL1 |  |  | 532 | 0.176 | 0.7928 | Yes |
| 290 | OLA1 |  |  | 536 | 0.175 | 0.7940 | Yes |
| 291 | NFIC |  |  | 543 | 0.174 | 0.7948 | Yes |
| 292 | FKBP3 |  |  | 547 | 0.173 | 0.7959 | Yes |
| 293 | FANCG |  |  | 554 | 0.172 | 0.7967 | Yes |
| 294 | CCT2 |  |  | 556 | 0.171 | 0.7981 | Yes |
| 295 | PPP1CA |  |  | 559 | 0.171 | 0.7993 | Yes |
| 296 | KRBOX1 |  |  | 562 | 0.170 | 0.8006 | Yes |
| 297 | HPF1 |  |  | 563 | 0.170 | 0.8020 | Yes |
| 298 | SUMO3 |  |  | 568 | 0.168 | 0.8030 | Yes |
| 299 | MAPRE1 |  |  | 569 | 0.168 | 0.8045 | Yes |
| 300 | BUB1B |  |  | 572 | 0.168 | 0.8057 | Yes |
| 301 | KIFC1 |  |  | 577 | 0.167 | 0.8067 | Yes |
| 302 | RMI2 |  |  | 579 | 0.166 | 0.8080 | Yes |
| 303 | PPIF |  |  | 580 | 0.166 | 0.8094 | Yes |
| 304 | PSMG2 |  |  | 581 | 0.166 | 0.8108 | Yes |
| 305 | TCTEX1D2 |  |  | 593 | 0.165 | 0.8110 | Yes |
| 306 | PARPBP |  |  | 594 | 0.164 | 0.8124 | Yes |
| 307 | POLE4 |  |  | 597 | 0.164 | 0.8136 | Yes |
| 308 | MCM8 |  |  | 601 | 0.163 | 0.8147 | Yes |
| 309 | NABP2 |  |  | 603 | 0.163 | 0.8160 | Yes |
| 310 | ECI1 |  |  | 607 | 0.162 | 0.8170 | Yes |
| 311 | CASP8AP2 |  |  | 608 | 0.162 | 0.8184 | Yes |
| 312 | VPS29 |  |  | 613 | 0.162 | 0.8194 | Yes |
| 313 | MCUR1 |  |  | 614 | 0.161 | 0.8207 | Yes |
| 314 | CBR3 |  |  | 616 | 0.161 | 0.8220 | Yes |
| 315 | RFC3 |  |  | 617 | 0.161 | 0.8234 | Yes |
| 316 | TUBB6 |  |  | 622 | 0.160 | 0.8243 | Yes |
| 317 | EIF4A3 |  |  | 635 | 0.158 | 0.8243 | Yes |
| 318 | E2F8 |  |  | 638 | 0.158 | 0.8255 | Yes |
| 319 | TIMM10 |  |  | 639 | 0.158 | 0.8268 | Yes |
| 320 | XRCC6 |  |  | 641 | 0.158 | 0.8281 | Yes |
| 321 | EXO1 |  |  | 643 | 0.157 | 0.8293 | Yes |
| 322 | TUBA1C |  |  | 644 | 0.157 | 0.8307 | Yes |
| 323 | SF3B4 |  |  | 649 | 0.156 | 0.8315 | Yes |
| 324 | GTF3A |  |  | 651 | 0.156 | 0.8328 | Yes |
| 325 | IER3IP1 |  |  | 652 | 0.156 | 0.8341 | Yes |
| 326 | PRIM1 |  |  | 660 | 0.155 | 0.8347 | Yes |
| 327 | NUDT5 |  |  | 663 | 0.155 | 0.8358 | Yes |
| 328 | PRIM2 |  |  | 667 | 0.154 | 0.8368 | Yes |
| 329 | AKR7A2 |  |  | 670 | 0.154 | 0.8379 | Yes |
| 330 | MCUB |  |  | 682 | 0.152 | 0.8379 | Yes |
| 331 | RPA1 |  |  | 687 | 0.151 | 0.8388 | Yes |
| 332 | TEDC2 |  |  | 695 | 0.151 | 0.8393 | Yes |
| 333 | WDR76 |  |  | 702 | 0.150 | 0.8399 | Yes |
| 334 | RBBP7 |  |  | 706 | 0.150 | 0.8409 | Yes |
| 335 | XRN2 |  |  | 710 | 0.149 | 0.8418 | Yes |
| 336 | ACADM |  |  | 715 | 0.148 | 0.8426 | Yes |
| 337 | PAXX |  |  | 716 | 0.148 | 0.8439 | Yes |
| 338 | PGK1 |  |  | 726 | 0.146 | 0.8442 | Yes |
| 339 | GINS4 |  |  | 727 | 0.146 | 0.8454 | Yes |
| 340 | RAD54L |  |  | 729 | 0.146 | 0.8466 | Yes |
| 341 | TRAP1 |  |  | 734 | 0.146 | 0.8474 | Yes |
| 342 | NFYB |  |  | 740 | 0.145 | 0.8481 | Yes |
| 343 | FANCA |  |  | 743 | 0.145 | 0.8491 | Yes |
| 344 | ITGB1BP1 |  |  | 750 | 0.144 | 0.8496 | Yes |
| 345 | ARPC5L |  |  | 767 | 0.142 | 0.8491 | Yes |
| 346 | EEF1B2 |  |  | 773 | 0.142 | 0.8497 | Yes |
| 347 | MNS1 |  |  | 774 | 0.142 | 0.8509 | Yes |
| 348 | CSNK2B |  |  | 786 | 0.140 | 0.8509 | Yes |
| 349 | RAD1 |  |  | 789 | 0.139 | 0.8519 | Yes |
| 350 | SKA3 |  |  | 790 | 0.139 | 0.8531 | Yes |
| 351 | CEP135 |  |  | 795 | 0.138 | 0.8538 | Yes |
| 352 | MMS22L |  |  | 799 | 0.138 | 0.8547 | Yes |
| 353 | PRTFDC1 |  |  | 818 | 0.137 | 0.8538 | Yes |
| 354 | GAMT |  |  | 825 | 0.135 | 0.8543 | Yes |
| 355 | EMP2 |  |  | 826 | 0.135 | 0.8555 | Yes |
| 356 | SMS |  |  | 846 | 0.133 | 0.8545 | Yes |
| 357 | CSRP2 |  |  | 847 | 0.133 | 0.8557 | Yes |
| 358 | MRPL37 |  |  | 855 | 0.132 | 0.8560 | Yes |
| 359 | PSMA3 |  |  | 866 | 0.131 | 0.8560 | Yes |
| 360 | TFDP1 |  |  | 874 | 0.130 | 0.8563 | Yes |
| 361 | ENOPH1 |  |  | 880 | 0.130 | 0.8569 | Yes |
| 362 | PARP2 |  |  | 886 | 0.129 | 0.8575 | Yes |
| 363 | ZNF367 |  |  | 896 | 0.128 | 0.8576 | Yes |
| 364 | EEF1E1 |  |  | 903 | 0.128 | 0.8580 | Yes |
| 365 | NRGN |  |  | 904 | 0.128 | 0.8591 | Yes |
| 366 | NCAPH2 |  |  | 910 | 0.127 | 0.8596 | Yes |
| 367 | DONSON |  |  | 929 | 0.125 | 0.8587 | Yes |
| 368 | ATP5MC1 |  |  | 935 | 0.125 | 0.8592 | Yes |
| 369 | SNX5 |  |  | 942 | 0.125 | 0.8596 | Yes |
| 370 | AK2 |  |  | 948 | 0.124 | 0.8601 | Yes |
| 371 | MRPS11 |  |  | 953 | 0.123 | 0.8607 | Yes |
| 372 | TALDO1 |  |  | 954 | 0.123 | 0.8618 | Yes |
| 373 | AHCY |  |  | 959 | 0.123 | 0.8624 | Yes |
| 374 | FARSB |  |  | 975 | 0.122 | 0.8617 | Yes |
| 375 | MZT2A |  |  | 977 | 0.122 | 0.8627 | Yes |
| 376 | IMMT |  |  | 988 | 0.121 | 0.8626 | No |
| 377 | NR2C2AP |  |  | 1005 | 0.120 | 0.8618 | No |
| 378 | ASRGL1 |  |  | 1011 | 0.119 | 0.8623 | No |
| 379 | RCC1 |  |  | 1021 | 0.118 | 0.8623 | No |
| 380 | DPM2 |  |  | 1046 | 0.116 | 0.8606 | No |
| 381 | CEP152 |  |  | 1047 | 0.116 | 0.8616 | No |
| 382 | MTFR2 |  |  | 1058 | 0.114 | 0.8615 | No |
| 383 | WDHD1 |  |  | 1092 | 0.112 | 0.8587 | No |
| 384 | MINOS1 |  |  | 1097 | 0.112 | 0.8593 | No |
| 385 | ACYP1 |  |  | 1109 | 0.111 | 0.8590 | No |
| 386 | RAC3 |  |  | 1129 | 0.110 | 0.8578 | No |
| 387 | ARL6IP6 |  |  | 1134 | 0.109 | 0.8583 | No |
| 388 | NDUFB11 |  |  | 1159 | 0.107 | 0.8565 | No |
| 389 | CCND3 |  |  | 1164 | 0.107 | 0.8570 | No |
| 390 | LRRCC1 |  |  | 1170 | 0.106 | 0.8574 | No |
| 391 | CENPJ |  |  | 1192 | 0.105 | 0.8559 | No |
| 392 | RDX |  |  | 1214 | 0.104 | 0.8545 | No |
| 393 | CISD2 |  |  | 1219 | 0.103 | 0.8549 | No |
| 394 | CTPS1 |  |  | 1223 | 0.103 | 0.8554 | No |
| 395 | SRPK1 |  |  | 1240 | 0.102 | 0.8545 | No |
| 396 | OLFM1 |  |  | 1263 | 0.101 | 0.8529 | No |
| 397 | JPT2 |  |  | 1273 | 0.100 | 0.8528 | No |
| 398 | C5orf34 |  |  | 1282 | 0.099 | 0.8528 | No |
| 399 | CLIC1 |  |  | 1291 | 0.099 | 0.8527 | No |
| 400 | FDPS |  |  | 1297 | 0.098 | 0.8530 | No |
| 401 | ETFB |  |  | 1334 | 0.096 | 0.8498 | No |
| 402 | KLHL23 |  |  | 1342 | 0.095 | 0.8498 | No |
| 403 | FAF1 |  |  | 1373 | 0.094 | 0.8473 | No |
| 404 | NUDT21 |  |  | 1379 | 0.093 | 0.8475 | No |
| 405 | MYBL1 |  |  | 1390 | 0.093 | 0.8472 | No |
| 406 | CHAC2 |  |  | 1392 | 0.093 | 0.8479 | No |
| 407 | RECQL4 |  |  | 1427 | 0.091 | 0.8449 | No |
| 408 | SHMT2 |  |  | 1449 | 0.090 | 0.8433 | No |
| 409 | MCMBP |  |  | 1451 | 0.089 | 0.8440 | No |
| 410 | SVIP |  |  | 1454 | 0.089 | 0.8445 | No |
| 411 | PIGX |  |  | 1522 | 0.085 | 0.8378 | No |
| 412 | FAM161A |  |  | 1523 | 0.085 | 0.8385 | No |
| 413 | CHAF1B |  |  | 1530 | 0.085 | 0.8385 | No |
| 414 | CCP110 |  |  | 1554 | 0.084 | 0.8367 | No |
| 415 | RBL1 |  |  | 1569 | 0.083 | 0.8358 | No |
| 416 | TMEM54 |  |  | 1591 | 0.082 | 0.8342 | No |
| 417 | PMVK |  |  | 1595 | 0.082 | 0.8346 | No |
| 418 | SRR |  |  | 1600 | 0.082 | 0.8348 | No |
| 419 | PAQR4 |  |  | 1601 | 0.082 | 0.8355 | No |
| 420 | PSMD14 |  |  | 1627 | 0.080 | 0.8334 | No |
| 421 | PRADC1 |  |  | 1653 | 0.079 | 0.8313 | No |
| 422 | RAB23 |  |  | 1663 | 0.079 | 0.8310 | No |
| 423 | CDCA7 |  |  | 1684 | 0.078 | 0.8294 | No |
| 424 | MTHFD1 |  |  | 1692 | 0.077 | 0.8293 | No |
| 425 | AUNIP |  |  | 1703 | 0.077 | 0.8288 | No |
| 426 | MACROD1 |  |  | 1768 | 0.074 | 0.8223 | No |
| 427 | NUP85 |  |  | 1772 | 0.074 | 0.8226 | No |
| 428 | LRRC45 |  |  | 1780 | 0.073 | 0.8225 | No |
| 429 | CDC7 |  |  | 1846 | 0.070 | 0.8158 | No |
| 430 | WDR54 |  |  | 1909 | 0.068 | 0.8095 | No |
| 431 | SAAL1 |  |  | 1988 | 0.065 | 0.8013 | No |
| 432 | CCDC15 |  |  | 2017 | 0.064 | 0.7988 | No |
| 433 | NFATC2IP |  |  | 2069 | 0.062 | 0.7936 | No |
| 434 | TCEAL9 |  |  | 2086 | 0.062 | 0.7923 | No |
| 435 | PSMB9 |  |  | 2093 | 0.061 | 0.7922 | No |
| 436 | GGH |  |  | 2149 | 0.059 | 0.7866 | No |
| 437 | PRKDC |  |  | 2154 | 0.059 | 0.7866 | No |
| 438 | ARRB2 |  |  | 2174 | 0.058 | 0.7850 | No |
| 439 | OIP5 |  |  | 2175 | 0.058 | 0.7855 | No |
| 440 | METRN |  |  | 2177 | 0.058 | 0.7859 | No |
| 441 | NUDT15 |  |  | 2188 | 0.058 | 0.7853 | No |
| 442 | CHTF18 |  |  | 2303 | 0.054 | 0.7730 | No |
| 443 | SCCPDH |  |  | 2482 | 0.048 | 0.7535 | No |
| 444 | LY6E |  |  | 2588 | 0.045 | 0.7422 | No |
| 445 | CNPY3 |  |  | 2607 | 0.044 | 0.7406 | No |
| 446 | KDELC2 |  |  | 2632 | 0.043 | 0.7383 | No |
| 447 | IL17RB |  |  | 2712 | 0.041 | 0.7298 | No |
| 448 | INAFM1 |  |  | 2731 | 0.041 | 0.7282 | No |
| 449 | RBBP4 |  |  | 2756 | 0.040 | 0.7258 | No |
| 450 | HAUS2 |  |  | 2827 | 0.038 | 0.7183 | No |
| 451 | TMEM237 |  |  | 2862 | 0.037 | 0.7149 | No |
| 452 | FBXO4 |  |  | 2891 | 0.037 | 0.7120 | No |
| 453 | MMD |  |  | 3023 | 0.033 | 0.6977 | No |
| 454 | TMEM107 |  |  | 3052 | 0.033 | 0.6949 | No |
| 455 | PFKP |  |  | 3170 | 0.030 | 0.6821 | No |
| 456 | TMUB1 |  |  | 3225 | 0.029 | 0.6763 | No |
| 457 | VKORC1 |  |  | 3270 | 0.028 | 0.6716 | No |
| 458 | SPATA33 |  |  | 3334 | 0.027 | 0.6648 | No |
| 459 | NRM |  |  | 3486 | 0.024 | 0.6482 | No |
| 460 | ABHD3 |  |  | 3775 | 0.019 | 0.6162 | No |
| 461 | C21orf58 |  |  | 3849 | 0.018 | 0.6082 | No |
| 462 | FADS1 |  |  | 3941 | 0.016 | 0.5982 | No |
| 463 | HMGA1 |  |  | 4135 | 0.013 | 0.5767 | No |
| 464 | TMEM14C |  |  | 4468 | 0.008 | 0.5397 | No |
| 465 | PIDD1 |  |  | 5535 | -0.009 | 0.4208 | No |
| 466 | PANK2 |  |  | 5559 | -0.009 | 0.4183 | No |
| 467 | PLP2 |  |  | 5612 | -0.010 | 0.4126 | No |
| 468 | VOPP1 |  |  | 6653 | -0.028 | 0.2968 | No |
| 469 | TMX1 |  |  | 6847 | -0.033 | 0.2755 | No |
| 470 | DDB2 |  |  | 7419 | -0.048 | 0.2122 | No |
| 471 | SLC20A1 |  |  | 7482 | -0.050 | 0.2057 | No |
| 472 | SCARA3 |  |  | 7514 | -0.051 | 0.2026 | No |
| 473 | ARPC1B |  |  | 7700 | -0.056 | 0.1825 | No |
| 474 | TECR |  |  | 7810 | -0.060 | 0.1708 | No |
| 475 | IKBIP |  |  | 8028 | -0.068 | 0.1472 | No |
| 476 | FUCA2 |  |  | 8068 | -0.070 | 0.1434 | No |
| 477 | KDELR2 |  |  | 8324 | -0.084 | 0.1157 | No |
| 478 | DEGS1 |  |  | 8459 | -0.093 | 0.1015 | No |
| 479 | PGRMC1 |  |  | 8958 | -0.143 | 0.0472 | No |
| 480 | CD151 |  |  | 9094 | -0.165 | 0.0335 | No |
| 481 | HACD3 |  |  | 9095 | -0.165 | 0.0349 | No |
| 482 | P4HB |  |  | 9115 | -0.169 | 0.0342 | No |
| 483 | CALU |  |  | 9278 | -0.223 | 0.0181 | No |
Table: GSEA details [plain text format]

  

Fig 2: DOROSZKO\_U3180MG\_CLUSTER\_3: Random ES distribution      
 Gene set null distribution of ES for **DOROSZKO\_U3180MG\_CLUSTER\_3**
